# Assessment of Patient Satisfaction Among Cancer Patients Undergoing Radiotherapy

**DOI:** 10.1007/s13187-020-01950-8

**Published:** 2021-01-11

**Authors:** R. Samant, E. Cisa-Paré, K. Balchin, J. Renaud, L. Bunch, P. Wheatley-Price, A. McNeil, S. Murray, J. Meng

**Affiliations:** The Ottawa Hospital Cancer Centre, 501 Smyth Road, Ottawa, Ontario K1H 8L6 Canada

**Keywords:** Patient satisfaction, HCP interaction, Patient-centered care

## Abstract

The patient-provider relationship is a key driver of patient satisfaction as it relates to overall healthcare experience. We surveyed patients undergoing radiation therapy to determine what they consider to be the most valued qualities in their interactions with the healthcare team. An ethics-approved 35-item patient satisfaction survey was developed in-house to gain insights on patients’ perception of their relationship with the healthcare team throughout their cancer journey. There were 199 completed survey, median age 68 years, 54% women and 45% men. Almost all (95%) “agreed” or “strongly agreed” that their physicians had been sensitive and compassionate. Over 90% felt that they received adequate explanations about their treatment, and had their questions answered. The vast majority (93%) felt included in the decision-making process. Patients reported the 5 most highly rated qualities among their healthcare providers (HCPs) as knowledge, kindness, honesty, good communication, and a cheerful attitude. Overall satisfaction was high but areas for improvement were identified including being offered future appointments for further discussion, more information about clinical trials, other treatments, and community resources. Patients noted their HCPs tended to focus on the physical and emotional needs of patients, but spiritual and cultural needs were rarely addressed. Patients receiving radiotherapy reported high rates of satisfaction across many aspects of their care. These findings also reinforce the different aspects of holistic care that can be improved, and serve as a reminder to clinicians that patients perceive their role as more than just that of a medical expert.

## Introduction

One of the main goals of healthcare providers (HCPs) in their interactions with patients is to help them feel better and to improve their quality of life. Patients’ wellness is the “raison d’être” of the entire medical system. Hence, a patient-centered model of care has been a healthcare system goal for decades now [1]. Measuring patient satisfaction drivers is an important metric in evaluating the quality of patient care provided by healthcare institutions [2–4].

There are many aspects to patient satisfaction, some of which are under the control of healthcare providers and some of which are not [5, 6]. Evidently, the outcome of a diagnosis and its treatment does influence how a patient feels, and this will be quite variable [7]. For example, a patient cured of his/her cancer while experiencing minimal side effects will almost certainly feel better than someone whose cancer has recurred and is now experiencing symptoms with clinical deterioration being inevitable. However, HCPs can influence how patients feel, and studies have been published indicating which qualities and attributes are valued most [8–10].

The incidence of cancer is increasing and it has become a leading cause of morbidity and mortality in North America. Cancer is also recognized as a growing problem worldwide. Treatment options including surgery, radiation, chemotherapy, immunotherapy, and other types of novel treatments continue to require extensive resources to meet patient needs. Having access to comprehensive cancer care involves much more than facilities and treatment options. The relationship between patients and providers plays a significant role in patient experiences. And this relationship now extends beyond the traditional nurse or physician rapport, to include allied health professionals and clerical and support staff. There are many ways healthcare professionals can improve the cancer patient experience, and this has been evaluated and published in a variety of settings [10–12]. It appears as though patient preferences will be influenced by a variety of factors and these are also influenced by the healthcare options available to them [8, 9].

Radiation therapy services are an integral part of a multi-disciplinary cancer program. In fact, around 50% of patients receive radiation treatment as part of their treatment journey. Here, we decided to evaluate patient satisfaction among those patients receiving radiation therapy. Patient satisfaction surveys among radiotherapy patients have been performed and published in the past, often for quality assurance purposes, usually assessing factors such as appointment times, appearance of the facilities, and friendliness of the staff [13–16]. In order to complement surveys administered in the past, we chose to focus this one on how patients perceived the quality of the interactions they had with their physicians and other HCPs. We specifically wanted to highlight what seemed to be the most valued HCP characteristics, and identify unmet patient needs. We hope to provide these insights to our multi-disciplinary team for the purpose of self-improvement and program enhancements and add to the body of research in the field of patient-centered care.

## Methods

An ethics-approved 35-item patient satisfaction questionnaire evaluating patient experience among cancer patients undergoing radiotherapy was developed by an interdisciplinary team of HCPs working within the radiation medicine program at The Ottawa Hospital Cancer Center. The questionnaire was reviewed by physicians, nurses, radiation therapists, and administrators. It evaluated a variety of domains with respect to the care patients received at the cancer center and the HCP qualities thought to be most important, including patient-physician interactions, the role of humor, and relationships with other HCPs. It was an anonymous, voluntary, and paper-based survey meant for self-completion requiring approximately 10 min. It was available in both official languages recognized by the hospital and administered to outpatients receiving radiation treatments.

A cross-sectional study design approach was used in the outpatient setting. Of the approximately 300 patients undergoing radiotherapy at the time of survey administration, the goal was to have 200 of them complete a survey. Patients were approached twice over a 5-month period to complete the survey over 1 week in November 2018 and again in February 2019. The main objectives of this research project were to evaluate how patients perceived the care they were receiving and to assess which qualities they valued most among their HCPs.

The survey responses were collated in an Excel spreadsheet, and descriptive statistics were calculated to generate the results. A series of preliminary analyses were conducted to identify correlations between patient satisfaction variables and demographic variables such as age, sex, cancer type, and time from cancer diagnosis. Bi-variate statistical tests such as chi-square and the Mann-Whitney *U* tests were used to determine possible associations. However, most tests did not meet the minimum sample size requirement to report results; therefore, *p* values were reported sparingly. Descriptive statistics (frequencies, percentages, medians, and means) were used instead. Subgroup analyses were conducted for the demographic variable of gender only as sample sizes from other variables were too small to conduct further analyses.

Included in the satisfaction questionnaire was a section where patients could provide comments with free text. Responses were analyzed using content analysis methods which aimed to identify common themes.

## Results

A total of 199 patients completed the survey. This represented approximately 30 to 35% of patients on radiotherapy during the survey period. The median age of respondents was 68 years, with 54% women and 45% men (1% unreported). Table 1 shows the distribution of patients, and the vast majority (85%) had been diagnosed within the past year. Responses to questions regarding the approach physicians used are shown in Table 2. The majority felt the approach of their physicians was appropriate, specifically with respect to conventionally accepted norms regarding physician-patient communication, including making eye contact and having friendly body language. Areas of improvement were noted as such: only 45% of patients felt they were informed of the availability of clinical trials and other treatment options (65%), along with information about psychosocial services (67%) and community resources (69%). Some patients (9%) also indicated that future appointments to review and discuss treatment were not offered. Most patients (93%) felt included in the decision-making process.

**Table 1:** Demographics of study population (*N* = 199)

Variable		Frequency	%
Sex	Female	108	54
	Male	89	45
	Unreported	2	1
Age (years)	Mean	66	
	40–49	12	6
	50–59	37	19
	60–69	53	27
	70–79	56	28
	> = 80	22	11
	Unreported	16	8
Cancer type	Breast	58	29
	Prostate	35	18
	Lung	29	15
	Head and neck	19	10
	Gynecological	17	9
	Other	38	19
	Unreported	4	2
Time from cancer Dx	Less than 1 year ago	170	85
	2–5 years ago	21	11
	6–10 years ago	3	2
	More than 10 years ago	3	2
	Unreported	2	1

**Table 2:** Summary of patient responses to questions regarding their perception of physician interaction using a 5-point Likert scale

When thinking about your recent visit(s) with your physician, how much do you agree/disagree with the following statements?	Strongly Disagree	Disagree	Neutral	Agree	Strongly Agree	Total
My physician explained my diagnosis in a sensitive manner.	2	0	7	62	128	199
	(1%)	(0%)	(4%)	(31%)	(64%)	(100%)
My physician has been compassionate in his/her approach when discussing my diagnosis.	1	1	8	63	126	199
	(1%)	(1%)	(4%)	(32%)	(63%)	(100%)
My physician properly explained the goals of my treatment.	0	0	6	72	119	197
	(0%)	(0%)	(3%)	(37%)	(60%)	(100%)
My physician properly explained the types of treatment (i.e., surgery, chemotherapy, radiation therapy).	0	2	7	59	130	198
	(0%)	(1%)	(4%)	(30%)	(66%)	(100%)
I understood the risks, benefits, and side effects of treatment.	0	4	6	75	107	192
	(0%)	(2%)	(3%)	(39%)	(56%)	(100%)
My physician informed me of other options for treatment.	2	20	43	50	69	184
	(1%)	(11%)	(23%)	(27%)	(38%)	(100%)
My physician informed me of clinical trials (if available).	10	28	57	32	45	172
	(6%)	(16%)	(33%)	(19%)	(26%)	(100%)
My physician provided enough time to answer my questions.	1	4	2	68	124	199
	(1%)	(2%)	(1%)	(34%)	(62%)	(100%)
My physician considered my values and concerns when discussing my treatment.	0	3	12	66	116	197
	(0%)	(2%)	(6%)	(34%)	(59%)	(100%)
My physician offered other appointments to discuss my diagnosis and treatment further if required.	1	16	39	58	80	194
	(1%)	(8%)	(20%)	(30%)	(41%)	(100%)
My physician offered contact information for community resources such as support groups (if requested).	1	14	40	56	69	180
	(1%)	(8%)	(22%)	(31%)	(38%)	(100%)
My physician offered me contact information for psychosocial services (if requested).	0	10	49	51	71	181
	(0%)	(6%)	(27%)	(28%)	(39%)	(100%)
My physician told me what the next step in my care would be.	1	3	17	84	84	189
	(1%)	(2%)	(9%)	(44%)	(44%)	(100%)
I felt included in the decision-making process.	0	3	11	78	100	192
	(0%)	(2%)	(6%)	(41%)	(52%)	(100%)
I know who to contact with any concerns I have about my illness.	0	6	17	69	105	197
	(0%)	(3%)	(9%)	(35%)	(53%)	(100%)
He/she uses appropriate eye contact.	0	0	5	65	127	197
	(0%)	(0%)	(3%)	(33%)	(64%)	(100%)
He/she includes friends/family members in the discussion.	0	0	17	53	123	193
	(0%)	(0%)	(9%)	(27%)	(64%)	(100%)
His/her body language is friendly and encouraging.	0	2	6	58	131	197
	(0%)	(1%)	(3%)	(29%)	(66%)	(100%)
He/she listens carefully to what I have to say.	0	1	4	57	136	198
	(0%)	(1%)	(2%)	(29%)	(69%)	(100%)

Table 3 highlights the importance of communication skills including having a positive attitude, listening carefully, and providing hope. The vast majority (> 85%) also indicated that smiling and using humor were important to patients.

**Table 3:** Summary of patient responses to questions regarding their perception of physician interaction using a 5-point Likert scale

When thinking about your interactions with staff, how important is it for them to:	Not at all important	Slightly important	Somewhat important	Very Important	Total
Use appropriate humor during your visits?	9	13	61	111	194
	(5%)	(7%)	(31%)	(56%)	(98%)
Smile during your visit?	2	8	34	154	198
	(1%)	(4%)	(17%)	(78%)	(100%)
Laugh with you in an appropriate manner?	5	18	49	123	195
	(3%)	(9%)	(25%)	(62%)	(98%)
Have a positive attitude?	0	0	27	171	198
	(0%)	(0%)	(14%)	(86%)	(100%)
Use humor to help in decreasing your anxiety?	8	24	45	112	189
	(4%)	(12%)	(23%)	(57%)	(96%)
Give a sense of hope in a sensitive and realistic manner?	5	10	34	148	197
	(3%)	(5%)	(17%)	(75%)	(99%)
Listen carefully to your personal needs and respond appropriately?	1	2	32	164	199
	(1%)	(1%)	(16%)	(82%)	(100%)

**Fig. 1:**
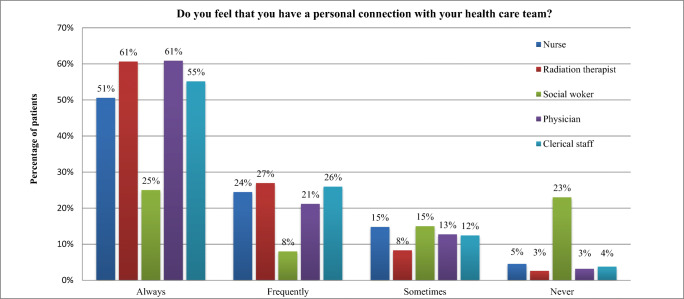
Patients rating of a personal connection with their healthcare providers using a 4-point Likert scale

**Fig. 2:**
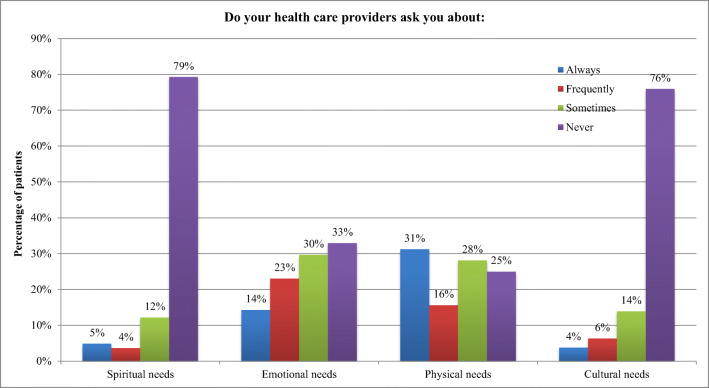
Patient rating of being asked by their healthcare providers regarding their various needs using a 4-point Likert scale

As shown in Fig. 1, most patients (72%) felt a personal connection to most of their HCPs. This connection appeared to be similar among most of the staff except for social workers. Eighty-eight percent of patients felt a personal connection with their radiation therapist, 82% with their radiation oncologist, 81% with the clerical staff, compared with only 33% with their social workers. Figure 2 shows that HCPs seemed to focus primarily on physical needs, and to a lesser degree on emotional needs. It seems not much attention was focused on the cultural and spiritual needs of patients, with 78% of patients stating that their HCPs never asked about these needs.

**Fig. 3:**
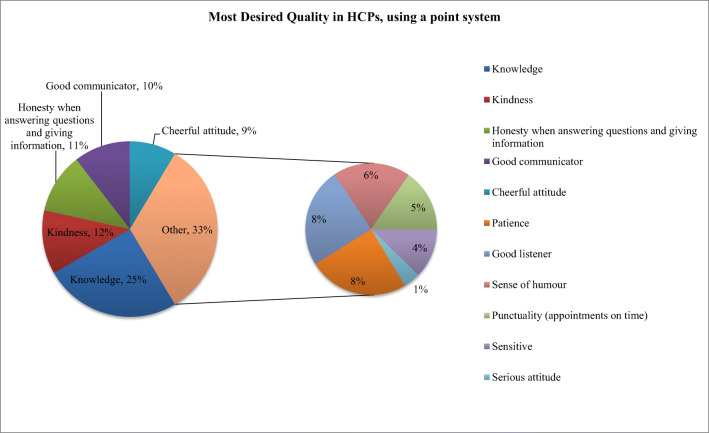
Patient ranking of most desired quality in their healthcare providers, using point system ranking

When asked to list the most valued qualities among their HCPs, knowledge was considered #1 most often (62%). Using a point system, we summed up all the scores for each quality and came up with a list of the most valued qualities listed by the respondents. The top five ranked qualities, in descending order of importance, were identified as knowledge, kindness, honesty, good communication, and a cheerful attitude. Figure 3 illustrates these rankings in graphical form. There was no statistically significant difference (*p* < 0.05) between responses from patients who identified as either male or female.

Of the 57 free text remarks left by patients, 49 were of a positive nature while only 8 included constructive criticism. A few of these were compiled (see Table 4). Staff consistency and scheduling of appointments were the most common complaints. Words that arose most within participants’ comments were kindness, compassion, and professionalism.

**Table 4:** Examples of patient remarks in reference to their satisfaction with the care received at the cancer center

Positive comments	Constructive criticism
“…You made my cancer journey easy and successful! I am forever grateful for your kindness and compassion…”	“Since staff change daily, there is no actual ‘team’...”
“I remain amazed by the professionalism of all staff at all levels…”	“…il devrait avoir plus de personnel qui parlent le français [en chimio]…”
“…all so kind and pleasant.”	“Try to coordinate […] timings for radiation so patients have less stress in coordinating their day lives.”
“Wonderful people, every one of them.”	“My appointment times keep changing without notification.”
“I would like to emphasize the extraordinary care I received [ …] highest degree of professionalism and appropriate level of empathy…”	“Patients need more listening from the oncologists and more time to answer all their questions..”

## Discussion

Patient satisfaction has been extensively studied both by researchers as well as healthcare organizations [3, 4, 8, 9, 17, 18]. There are many reasons for assessing satisfaction but the most important is to evaluate if the needs of patients are being met and to identify gaps and areas for improvement. A variety of trends are seen and they vary with patient population and healthcare systems, along with available resources and expectations. In North America, where healthcare resources are relatively abundant, the expectations appear quite high.

Our results among cancer patients appear to be consistent with the available published literature [2, 5, 7, 8, 10, 19]. Patients typically report high levels of satisfaction in surveys in relation to the quality of their care, their treatment, and the attitude of staff. Similarly, the respondents in our study generally appear to be satisfied with the care they received. The patient responses suggest a perceived need for more information about clinical trials, alternative treatment options availability, and the opportunity to schedule a follow-up appointment to finalize a decision to treat. In other published studies, patients have echoed the theme of wanting more information and the desire to have more time to discuss their individual situation with their HCPs. This clearly highlights the key role of education in developing management plans for patients.

Other areas for improvement included having more information about psychosocial and community resources, and support for cultural and spiritual needs. This trend has been reported in the literature, and possible explanations may include limited time and resources [5, 8, 16]. It appears as though cancer centers tend to mostly focus on direct medical needs and treatments of patients. As the cultural diversity of our communities continues to increase, more efforts are required to be socially accountable to our treatment populations. Resources need to be allocated for research and education to deliver care that is truly person-centered. For example, our cancer center recognized that the unique needs of our Indigenous peoples were often underappreciated. We therefore established a program, with guidance from Indigenous leaders, to provide culturally competent care in a way that compliments standard healthcare.

Resources strained due to budget limitations, time constraints, and availability of specialized HCPs were likely the reason for the few unfavorable remarks left by patients during our study. Although we strive for world-class care at our center, we consider scheduling conflicts to be somewhat minor. Notwithstanding these limitations, there are still many things that can and should be done to meet the needs of cancer patients. Some of the important factors that patients appreciate are related to effective communication and compassion shown by healthcare providers [5, 12, 20]. The way HCPs present themselves and interact with their patients can have a great impact on their experience. A positive attitude appears essential according to our respondents. Smiling, encouraging body language, and making eye contact standout as simple elements to incorporate in HCPs’ routine as they meet with patients. Patients also value the use of humor and hope during their interactions [21–23]. HCPs need to be made aware and constantly reminded how these traits can have a positive impact on patients’ perception of quality care.

The relationship between healthcare providers and cancer patients is important; therefore, developing a “connection” should be fostered [24]. Patients surveyed felt connected to most of their HCPs, except for social workers. This is believed to be due to the sporadic nature of patients’ contact with social workers compared to the frequent (often daily for up to 8 weeks) interactions with radiation therapists, nurses, clerks, and oncologists. We postulate that patients’ personal connections with HCPs are dependent upon the time spent with those respective HCPs, though sample sizes for this type of subgroup analysis rendered statistical tests invalid. It was enlightening to observe which qualities were valued most by patients. “Knowledge” was considered most important, but other valued qualities may not be as obvious to healthcare providers such as kindness, honesty, good communication, and a cheerful attitude. These findings appear consistent with other studies [5, 8, 10].

Stress and burnout among healthcare providers are increasingly being reported and it is known that this can negatively affect patient care [25, 26]. HCPs need to be aware of the impact their attitude has on patient-provider relationships. Encouraging feedback from patients could have a positive impact on job satisfaction among healthcare providers, possibly helping to reduce severity of stress and risk of burnout. Through education and training, HCPs can be reminded of what cancer patients value most and this can, and should, lead to better care [5, 6, 20]. HCPs cannot influence all aspects of healthcare delivery but should be aware of what they can do to make a difference.

There are limitations to our study. These include the fact that only 30 to 35% of patients on radiotherapy completed the survey, and this could be a source of bias among those who completed the survey. It is uncertain if they fairly represent all patients on treatment; one could argue that patients who are very satisfied with their care would be more likely to complete the survey, while an alternate argument could be that patients who are not satisfied with their experience would be more inclined to express their discontent by answering the survey. It is also known that patients often try to be positive in the responses given to surveys [2, 15] and usually do not like to criticize the care they receive, especially during treatment and within a socialized universal healthcare model where cancer care is essentially provided free of charge. This can be seen by the high volume of patients who selected a neutral response, especially for survey questions that were expected to have been poorly ranked. Also, most of the patients who responded to the survey were diagnosed within the previous year and may not reflect the opinions of those diagnosed with cancers for longer periods of time. All the responders were also receiving radiotherapy and it is uncertain if the responses would be similar among those receiving other types of treatment or after completion of active treatment. We also did not ask patients to specify whether the treatment they were receiving was curative (or palliative), and this could also influence responses related to patient satisfaction with care. Finally, we developed our own survey questions because of our specific areas of interest, and did not use one of the standardized published survey questionnaires [2, 5–7].

However, the results of the study are a starting point to remind healthcare providers which attributes and qualities cancer patients value most, and the improvement areas to focus on. It also highlights what patients believe are the most important aspects in the care they receive, especially with regard to education and resource information, which should be top of mind for the healthcare team. We have presented the results of our study to senior management and the goal is also to present this information to our cancer center staff. This type of information can be used by frontline HCPs, administrators, and educators to create and prioritize patient-centered educational tools, develop professional development courses, and evaluate the quality of services provided. We believe this can potentially improve job satisfaction among healthcare providers and hope to survey them in the future to test this hypothesis. Follow-up surveys should also be considered to ensure there is a process of continuous improvement.

## Conclusions

Patients undergoing radiation therapy at our institution reported high rates of satisfaction across many aspects of their care. However, they do indicate areas for improvement, including more information about treatment options and supportive care services. Also, the HCP qualities they value most in addition to knowledge and good communication include kindness, honesty, and a cheerful attitude. All of this reminds us of the importance of holistic care.

## References

[1] Loblaw DA, Bezjak A, Bunston T (1999). Development and testing of a visit-specific patient satisfaction questionnaire: the Princess Margaret Hospital satisfaction with doctor questionnaire. J Clin Oncol.

[2] Bredart A, Martinez M, Salgado E, Lainez N, Vera R (2012). The cancer outpatient satisfaction with care questionnaire for chemotherapy, OUT-PATSAT35 CT: a validation study for Spanish patients. Support Care Cancer.

[3] McDaniel C, Nash JG (1990). Compendium of instruments measuring patient satisfaction with nursing care. QRB Qual Rev Bull.

[4] Sitzia J, Wood N (1997). Patient satisfaction: a review of issues and concepts. Soc Sci Med.

[5] Lis CG, Rodeghier M, Gupta D (2009). Distribution and determinants of patient satisfaction in oncology: a review of the literature. Patient Prefer Adherence.

[6] Sandoval GA, Brown AD, Sullivan T, Green E (2006). Factors that influence cancer patients’ overall perceptions of the quality of care. Int J Qual Health Care.

[7] Nguyen TV, Anota A, Bredart A, Monnier A, Bosset JF, Mercier M (2014). A longitudinal analysis of patient satisfaction with care and quality of life in ambulatory oncology based on the OUT-PATSAT35 questionnaire. BMC Cancer.

[8] Wiggers JH, Donovan KO, Redman S, Sanson-Fisher RW (1990). Cancer patient satisfaction with care. Cancer.

[9] Oberst MT (1984). Methodology in behavioral and psychosocial cancer research. Patients’ perceptions of care. Measurement of quality and satisfaction. Cancer.

[10] Radwin L (2000). Oncology patients’ perceptions of quality nursing care. Res Nurs Health.

[11] Von Essen L, Sjoden PO (1991). Patient and staff perceptions of caring: review and replication. J Adv Nurs.

[12] Sherlaw-Johnson C, Datta P, McCarthy M (2008). Hospital differences in patient satisfaction with care for breast, colorectal, lung and prostate cancers. Eur J Cancer.

[13] French J, McGahan C (2009). Measuring patient satisfaction with radiation therapy service delivery. Healthc Manage Forum.

[14] Hashmi F, Gregor N, Liszewski B, Bola R, Kulczyski S, Nathoo D, Su H, Tirona R, Russell S, Turner A, Di Prospero L, D’Alimonte L, McGuffin M (2019). It only takes a minute: the development and implementation of a patient experience survey in radiation therapy. J Med Imaging Radiat Sci.

[15] Talamini R, Boz G, Franceschi S, Franchin G, Trovo MG (1991). Evaluation of hospital care in a radiotherapy department in north-eastern Italy. Eur J Cancer.

[16] Zissiadis Y, Provis A, Harper E, Kearney E, McDonald L, Dhaliwal S (2006). Patient satisfaction in radiation oncology. Australas Radiol.

[17] Carroll L, Sullivan FM, Colledge M (1998). Good health care: patient and professional perspectives. Br J Gen Pract.

[18] Coulter A (2002). Patients’ views of the good doctor. BMJ.

[19] Larson PJ (1984). Important nurse caring behaviors perceived by patients with cancer. Oncol Nurs Forum.

[20] Widmark-Petersson V, von Essen L, Sjoden PO (2000). Perceptions of caring among patients with cancer and their staff. Differences and disagreements. Cancer Nurs.

[21] Hunt AH (1993). Humor as a nursing intervention. Cancer Nurs.

[22] McClement SE, Chochinov HM (2008). Hope in advanced cancer patients. Eur J Cancer.

[23] Sanatani M, Schreier G, Stitt L (2008). Level and direction of hope in cancer patients: an exploratory longitudinal study. Support Care Cancer.

[24] Halldorsdottir S, Hamrin E (1997). Caring and uncaring encounters within nursing and health care from the cancer patient’s perspective. Cancer Nurs.

[25] Grunfield E, Whelan TJ, Zitzelsberger L, Willan AR, Montesanto B, Evans WK (2000). Cancer care workers in Ontario: prevalence of burnout, job stress and job satisfaction. CMAJ.

[26] Sehlen S, Vordermark D, Schafer C, Herschbach P, Bayeri A, Pigorsch S, Rittweger J, Dormin C, Bolling T, Wypior HJ, Zehentmayr F, Schulze W, Geinitz H (2009). Job stress and job satisfaction of physicians, radiographers, nurses and physicists working in radiotherapy: a multicenter analysis by the DEGRO Quality of Life Work Group. Radiat Oncol.

